# Folic Acid Induces Intake-Related Changes in the Mammary Tissue Transcriptome of C57BL/6 Mice

**DOI:** 10.3390/nu12092821

**Published:** 2020-09-15

**Authors:** Mark A. Burton, Elie Antoun, Reyna S. Penailillo, Graham C. Burdge, Karen A. Lillycrop

**Affiliations:** 1 School of Human Development and Health, Faculty of Medicine, University of Southampton, Southampton SO16 6YD, UK; m.a.burton@soton.ac.uk (M.A.B.); Elie.Antoun@soton.ac.uk (E.A.); g.c.burdge@soton.ac.uk (G.C.B.); 2 Institute of Developmental Sciences, Centre for Biological Sciences, Faculty of Natural and Environmental Sciences, University of Southampton, Southampton SO16 6YD, UK; reyna.penailillo@gmail.com

**Keywords:** folic acid, mammary gland, transcriptome, breast cancer, EZH2

## Abstract

Folic acid (FA) intake has been associated with increased breast cancer risk in some studies. Although underlying mechanisms are unknown, epigenetic modifications that persistently alter transcription have been suggested. We tested the hypothesis that high FA (HFA) intake alters the adult mammary transcriptome in a manner consistent with increased potential for carcinogenesis, detectable beyond the period of intake. C57BL/6 mice were fed control FA (CFA) (1 mg/kg diet) or HFA (5 mg/kg diet) diets for 4 weeks, followed by AIN93M maintenance diet for 4 weeks. Plasma 5-methyltetrahydrofolate, *p*-aminobenzoylglutamate and unmetabolised FA concentrations were greater (1.62, 1.56, 5.80-fold, respectively) in HFA compared to CFA mice. RNA sequencing of the mammary transcriptome (~20 million reads) showed 222 transcripts (191 upregulated) differentially expressed between groups. Gene Set Enrichment showed upregulated genes significantly enriched in Epithelial Mesenchymal Transition, Myogenesis and Apical Junction and downregulated genes in E2F targets, MYC targets and G2M checkpoint. Cancer was the most altered Disease and Disorder pathway, with Metastasis, Mammary Tumour and Growth of Tumour the most upregulated pathways. ChIP-seq enrichment analysis showed that targets of histone methyltransferase EZH2 were enriched in HFA mice. This study demonstrates HFA intake during adulthood induces mammary transcriptome changes, consistent with greater tumorigenic potential.

## 1. Introduction

Global folic acid (FA) intake has increased [1] due, in part, to the mandatory fortification of foods, the general increase in use of supplemental FA, and peri-conceptional FA supplementation recommendations for women during conception and pregnancy, to prevent the development of neural tube defects [2]. However, there is evidence that folate and its synthetic form FA can influence cancer development and progression [3,4]. For example, some studies [5,6,7,8,9], although not all [10,11], have shown that dietary folate intake or blood folate concentration are inversely related to the risk of developing some cancers including breast, colorectal, ovarian, bladder and prostate. In contrast, higher FA intake has been suggested to increase the risk of breast cancer (BC) [6,12,13]. For example, the Prostate, Lung, Colorectal, and Ovarian Cancer Screening Trial showed an increased risk (19%) of BC in postmenopausal women after FA supplementation (≥400 µg/d), while a meta-analysis of 16 prospective studies that involved 744,068 participants reported a U-shaped relationship between dietary total folate intake (which includes FA) and BC risk [13]. In this study, women with daily dietary folate intake between 153 and 400 μg had significantly reduced BC risk compared with those who consumed less than 153 μg or more than 400 μg.

Studies in animal models have also suggested that higher FA intake can promote cancer development, however, this effect may be dependent upon both the level of intake and the presence of preneoplastic lesions. For example, in mice, high FA (HFA) intake (5 mg FA/kg diet) promoted the progression of pre-existing 7, 12-dimethylbenz (a) anthracene (DMBA)-induced mammary tumours compared to mice fed a diet of 2 mg FA/kg [14]. The increase in mammary tumorigenesis was accompanied by an increase in the expression of human epidermal growth factor receptor 2 (HER2), whose amplification or over-expression has been shown to play an important role in the pathogenesis and progression of BC [15]. Maternal and postweaning FA supplementation (5 mg/kg HFA diet) have also been reported to increase the risk of mammary adenocarcinomas in offspring after DMBA exposure [16], suggesting that high maternal FA intake can alter long term cancer susceptibility in offspring. Conversely, Sie et al., reported that high maternal FA intake (5 mg/kg) during pregnancy and lactation reduced the number of terminal endbuds (the structures from which mammary tumours are initiated) in offspring in the absence of a carcinogen [16]. FA may, therefore, induce a modulatory effect on mammary gland development and subsequent tumorigenesis that persists after the period of supplementation and which is dependent upon the dose, stage of cell transformation and time of supplementation.

Tetrahydrofolate, a reduced form of FA, is a cofactor in the supply of methyl groups for both histone and DNA methylation reactions, the dysregulation of which has been implicated in both the initiation and progression of many cancers [17,18]. Therefore, one potential mechanism by which FA may influence BC risk is through alterations in epigenetic processes leading to changes in gene transcription that affect both short- and long-term cancer susceptibility in both mother and offspring. This is supported by findings that show increased global DNA hypomethylation and decreased DNA methyltransferase (*Dnmt1*) expression respectively in rodent offspring after maternal or post-weaning FA supplementation [16]. Although epigenetic processes have been suggested to be most sensitive to fluctuations in the intake of methyl donors and cofactors during early life, there is increasing evidence that there is considerable plasticity of the epigenome during adulthood [19]. However, despite the increased intake of FA worldwide through supplementation and food fortification in adult populations [20], few studies have investigated how HFA intake during adulthood affects the global mammary transcriptome.

To address this, we investigated whether increased FA intake during adulthood induces changes to the mammary transcriptome that are detectable after the period of supplementation with the aim of gaining novel insights into the underlying mechanisms by which HFA can modulate long-term mammary tumorigenesis.

## 2. Materials and Methods

### 2.1. Study Design

All mice and experimental procedures were conducted using protocols approved by, and in accordance with, the UK Home Office Animal (Scientific Procedures) Act 1986 at the University of Southampton under UK Home Office project license PPL30/2467 and local ethics committee (60985). Virgin female C57BL/6 mice supplied by Charles River UK were fed a maintenance diet (RM1; Special Diet Services, Ltd., Witham, UK) prior to the experimental period. On post-natal day (PND) 74, C57BL/6 mice (*n* = 10 per dietary group) were fed modifications of the AIN93M semi-purified diet (Test Diet™, St. Louis, MO, USA) containing either 1 mg FA/kg (control FA, CFA) diet, which is considered sufficient to meet the nutritional requirement for the adult laboratory mouse [21], or 5 mg FA/kg (high FA, HFA) diet for 4 weeks (PND 74–102) before being transferred to the AIN93M maintenance diet (Test Diet™) for a further 4 weeks (PND 102–130). The experimental period from PND 74–130 in mice corresponds to mature adulthood. The translation in age between humans and mice is complex, dependent on the period of development and can only be approximated. The maturation rate of a mouse at this stage of life has been estimated to be 45× that of a human, with the 4 week period of feeding the HFA diet equivalent to approximately 3.5 years in humans [22]. The nutrient composition of the diets is summarised in Supplementary Table S1. A total of 1 mg FA/kg of diet was selected to reflect the human RDA of 200 ug/day and 5 mg FA/kg diet was selected to reflect approximately 5× the human RDA (~0.8–1.2 mg FA/day), obtained through food fortification (~0.2–0.4 mg FA/day) and multivitamins/food supplements (~0.6–0.8 mg FA/day). Diets and water were provided ad libitum. All mice were fasted for 6 h prior to tissue collection. Mice were culled in line with methods described previously [23]. Blood was collected by cardiac puncture into a 1.2 mL collection tube using EDTA as an anticoagulant and centrifuged at 181× *g* for 10 min. Plasma was snap-frozen in liquid nitrogen to preserve FA/folate metabolites. There were 10 mice per dietary group used for RNA analysis, of which 6 were used for sequencing, and an additional 8 mice per dietary group were used for blood folate measurements. Right thoracic mammary gland fat pads were excised intact, snap-frozen in liquid nitrogen and stored at −80 °C until required.

### 2.2. Body Weights and Food Intake

Individual body weight and cage food intake over 24 h were measured weekly throughout the period of feeding the Test Diets (PND 74–102) and up to 4 weeks after the end of the dietary intervention (PND 102–130). Energy intake (kJ per 10g of mouse) was estimated for individual mice based on total food consumption over 24 h per cage (*n* = 10). Growth and energy intake were assessed by calculating the difference in area under the weight * time or energy intake * time curves (AUC). Briefly, AUC were calculated for each mouse and data from mice fed HFA or CFA were compared by unpaired Student’s unpaired *T*-tests. Average daily FA intake was estimated using whole cage intakes and the FA content of the diet.

### 2.3. Measurement of Plasma Folates and Folate Metabolites

The concentrations of folate metabolites 5-methyltetrahydrofolate (5-meTHF), the main active form of folate in plasma, 4-α-hydroxy 5-methyltetrahydrofolate (hmTHF), FA, 5-formyltrahydrofolate (5fTHF), and two catabolites of folate, namely p-aminobenzoylglutamate (pABG) and p-acetamidobenzoylglutamate (apABG), were measured at the end of the supplementation period (PND 102) using liquid chromatography-tandem mass spectrometry. The measurements were conducted by BEVITAL (Bergen, Norway).

### 2.4. RNA Isolation

RNA was extracted from frozen mammary glands using the mirVANA RNA Isolation Kit (Invitrogen, Waltham, MA, USA), following manufacturer’s instructions. RNA concentration and purity were obtained using a Nanodrop 1000 spectrophotometer (Thermo Scientific, Waltham, MA, USA). Samples (*n* = 6 per group) were also assessed for RNA quality and concentration using an Agilent Bioanalyser 2100 (Agilent Technologies, Santa Clara, CA, USA).

### 2.5. RNA-Seq

Total RNA-seq (~20 million reads, 75 bp paired end sequencing) was carried out by Oxford Gene Technology (Oxford, UK). Briefly, samples were prepared using the Illumina TruSeq RNA Sample Prep Kit v2 (Illumina, San Diego, CA, USA). Sequencing was performed on the Illumina HiSeq2000 platform using TruSeq v3 chemistry (Illumina, San Diego, CA, USA).

### 2.6. Bioinformatics Analysis

Raw FASTQ files were run through FASTQC (Babraham Institute, UK) to determine quality control (QC) metrics at the read level. One sample (5 mg/kg diet) had poor quality reverse reads and so was discarded from subsequent analysis. Reads were aligned to the current release of the mouse genome (GRCm38) in Ensembl using the TopHat aligner [24] (version 2.0.14) which utilises Bowtie [25] (version 2.2.5). Count tables were generated using HTSeq [26] (version 0.6.1). The reference gene transfer format file used with HTSeq was obtained from Ensembl for the mouse genome release GRCm 38.83. Mean absolute deviation (MAD) score analysis was carried out during QC, to determine outlier samples. One outlying sample based on the MAD score was removed from subsequent analysis and consequently 5 samples/group were used in the final analysis. The data set is available at the NCBI Gene Expression Omnibus (GEO) under accession GSE153732.

Count normalization and differential expression was carried out using the EdgeR package in R [27] (version 3.2.1). The group and cage of mice were included in the model. Genes were classed as differentially expressed if the false discovery rate (FDR) was < 0.05. Gene Set Enrichment Analysis (GSEA) was carried out using GSEA v2.2.2 from the Broad Institute (University of California, San Diego). The following gene set databases were used from the Molecular Signatures Database (MSigDB): hallmark gene sets (H), curated gene sets (C2) and gene ontology (GO) gene sets (C5). The GSEA Preranked tool (Broad Institute, University of California San Diego) was used, using a pre-ranked gene list provided to the software. Genes were ranked based on their fold change (FC) and nominal *p*-values. Chip-seq enrichment analysis was carried out using the Chip-seq significance tool (Encode, UK). Differentially expressed and background genes were entered and genes with enriched binding sites were analysed in the whole gene body, 500 bp upstream or 1000 bp downstream of the transcription start site (TSS, 5′End). Genes were compared against human mammary cell lines HMEC and MCF7.

### 2.7. Pathway Analysis

Pathway analysis was performed using Ingenuity Pathway Analysis software (IPA) (Qiagen, Hilden, Germany). Briefly, RNA-seq data was assessed for biological pathways with significance set at FDR < 0.05 and differential expression cut-offs at 0.5 FC.

### 2.8. Quantitative Real Time PCR (qRT-PCR)

Quantitative real time PCR (qRT-PCR) was carried out using a Light Cycler 480 real-time PCR System (Roche, Basel, Switzerland). A total of 1 μg total RNA extracted from the 2nd and 3rd thoracic mammary glands was DNase treated (Sigma-Aldrich, St Louis, MO, USA and reverse transcribed to cDNA using Moleney Murine Leukaemia reverse transcriptase (Promega, Madison, WI, USA). cDNA was amplified with commercial real-time qRT-PCR primers for *Mmp2* and *Mmp3* (Qiagen, Hilden, Germany) (Supplementary Table S2). qRT-PCR reactions were performed using Quantifast SYBER Green Master Mix (Qiagen, UK). Housekeeping genes optimised for mouse mammary tissue have been previously identified [28]. A panel of six genes using commercially available primers (Primer Design, Southampton, UK) was assessed using our samples by the GeNorm method [29]. Briefly, for each selected housekeeping gene the software determined the pairwise variation with all other control genes as the standard deviation of the logarithmically transformed expression ratios, and defined the internal control gene-stability measure *M* as the average pairwise variation of an individual gene with all other control genes [29]. Genes (*Gapdh* and *Ubc*) with the lowest *M* values (*M* < 0.6) demonstrated the most stable expression across treatments and were used to normalise Ct values from genes of interest. Samples were measured in duplicate and the overall change in expression calculated using standard curve analysis [30].

### 2.9. Statistical Analysis

Student’s *T*-test, correlation analysis, AUC analysis and Benjamini–Hochberg analysis were performed using SPSS Statistics (IBM, Version 22) and Graph Pad Prism (Version 5.0). *p* values less than 0.05 were considered statistically significant.

## 3. Results

### 3.1. Higher FA Intake Did Not Alter Body Weight or Energy Intake

There were no significant differences between dietary groups in body weight (g * day) or energy intake (kJ per 10 g of mouse * day) during the four week period of supplementation (PND 74–102) or during the following four week period (PND102–130) when mice were fed the maintenance diet (Supplementary Table S3). Average FA intake (24 h) during the supplementation period (PND 74–102) estimated that mice fed CFA consumed 2.6 µg FA, compared with mice fed the HFA diet who consumed 13.3 µg FA.

### 3.2. Higher FA Intake Increased Folate Metabolite and Catabolite Concentrations in Plasma

Plasma total FA, folate and metabolites were measured at the end of the supplementation period on PND 102. 5-meTHF, p-aminobenzoylglutamate (pABG), a product of folate degradation, and unmetabolised FA concentrations were significantly higher (1.62, 1.56 and 5.80-fold, respectively) in the HFA group compared to the CFA group. There was no statistically significant difference in p-acetamidobenzoylglutamate (apABG) or 5-formyltetrahydrofolate, (5-fTHF) concentrations between dietary groups (Table 1).

### 3.3. Higher FA Intake Induced Changes in the Mammary Gland Transcriptome

Total RNA-seq (20 million reads, 75PE) was carried out on RNA extracted from the mammary glands of adult mice, 4 weeks after the end of the FA supplementation period on PND 130. Comparison of gene expression profiles identified 222 differentially expressed (FDR adjusted *p* < 0.05) transcripts, of which 191 were upregulated (Supplementary Table S4). The top three significantly upregulated transcripts (FDR adjusted *p* < 0.05) were Dynamin 1 (*Dnm1*) (FDR, 9.71 × 10^−6^), CD209g antigen (*Cd209g*) (FDR, 9.80 × 10^−6^) and Gamma-Aminobutyric Acid (GABA) A Receptor, Subunit Alpha 3 (*Gabra3*) (FDR, 1.24 × 10^−5^) (Table 2). The top three significantly downregulated transcripts were Bcl2 Binding Component 3 (*Bbc3*) (FDR, 1.66 × 10^−4^), Small Nucleolar RNA Host Gene 12 (*Snhg12*) (FDR, 8.11 × 10^−4^) and Ribosomal Protein L22 Like 1 (*Rpl22l1*) (FDR, 8.885 × 10^−4^) (Table 2).

### 3.4. Gene Ontology and Gene Set Enrichment Analysis Identified Differentially Expressed Gene Sets in Response to Increased FA Intake

Gene ontology (GO) analysis was performed to investigate the possible functional significance of the changes in the transcriptome. The pathways most significantly enriched in the Biological Process category were locomotion (FDR, 3.50 × 10^−13^), cell migration (FDR, 1.30 × 10^−12^) and cell motility (FDR, 2.70 × 10^−12^). The top five differentially expressed genes in each of these pathways were the same, namely *Ackr3*, *C5ar1*, *Itga11*, *Cd248* and *Cc19*. The three most enriched pathways in the molecular function category and the top five genes differentially expressed in each pathway were collagen binding (FDR, 7.80 × 10^−7^) (*Col6a2*, *Itga11*, *Mrc2*, *Col6a1*, *Tnxb*), metallopeptidase activity (FDR, 1.60 × 10^−6^) (*Ace*, *Mmp9*, *Naalad2*, *Timp2*, *Timp3)* and glycosaminoglycan binding (FDR, 3.00 × 10^−6^) *(Pf4*, *Tnxb*, *Pcolce*, *Fnl*, *Col5a1*). The top three pathways enriched in the cellular compartment category and top five genes differentially expressed in each pathway were extracellular matrix (ECM) (1.20 × 10^−15^) (*Col6a2*, *Col6a3*, *Pf4*, *Col6a1*, *Emilin2*), extracellular space (FDR, 9.40 × 10^−14^) (*Col6a2*, *Col6a3*, *Ccl9*, *Pf4*, *Col6a1*) and extracellular region *(Col6a2*, *Col6a3*, *Ccl9*, *Sned1*, *Col163*) (Supplementary Table S5).

Gene Set Enrichment Analysis (GSEA) was also performed to identify over- and underrepresented gene sets amongst the differentially expressed genes. Hallmark gene sets, which represent specific, well-defined biological processes based on identifying overlaps between gene sets in MSigDB collections, and C2 curated gene sets, created from online pathway databases, were examined. The top three significantly enriched Hallmark gene sets (Supplementary Table S6) that were upregulated in response to increased FA intake were Epithelial Mesenchymal Transition (EMT) (Normalized Enrichment Score (NES) = 7.84, FDR < 0.001) (Supplementary Table S7), Myogenesis (NES = 6.92, FDR < 0.001) and Apical Junction (NES = 7.84, FDR < 0.001). The top three significantly downregulated enriched gene sets were E2F targets (NES = −6.67, FDR < 0.001) (Supplementary Table S8), MYC targets (NES = −4.81, FDR < 0.001) and G2M checkpoint (NES = −4.37, FDR < 0.001), Within the C2 category, the top three significantly upregulated enriched gene sets were Naba Matrisome (NES = 11.47, FDR < 0.001), Lim Mammary Stem Cell Up (NES = 10.64, FDR < 0.001) and Naba Core Matrisome (NES = 9.12, FDR < 0.001). The top three significantly downregulated enriched gene sets were Pujana BRCA2 Pcc Network (NES = −7.10, FDR < 0.001), Marson by E2F4 Unstimulated (NES = −6.24, FDR < 0.001) and Reactome Processing of Capped Intron Containing Pre MRNA (NES = −5.94, FDR < 0.001) (Supplementary Table S9).

### 3.5. Pathway Analysis Identified Disease Pathways Linked to Breast Cancer and Metastasis

Ingenuity Pathway Analysis was performed to gain further insights into the disease pathways and to identify the upstream regulators affected by increased FA intake. Cancer was the most significantly affected disease pathway (*p* = 8.09 × 10^−3^–4.00 × 10^−10^) in which 56 genes were altered (Supplementary Table S10). Within the cancer pathway, the top three most activated pathways were metastasis (Z = 1.206), mammary tumour (Z = 1.118) and growth of tumour (Z = 0.592) (Supplementary Figure S2A,B). Eleven genes were differentially regulated in the metastasis pathway, namely *Fn1*, *C5ar1*, *Mmp3*, *Mmp9*, *Il1rl1*, *Ednrb*, *Hic1*, *Akap12*, *Ret*, *Malat1 and Ifitm3*. Top networks were identified as cell morphology, cellular assembly and organization and cellular function and maintenance (Supplementary Table S11). Alpha catenin (*Ctnna1*) was identified as the top upstream regulator (*p* = 1.39 × 10^−20^) which was predicted, based on its interaction pathway, to be inhibited in the HFA group (Supplementary Figure S2C and Table S12). Additional upstream regulators were *Il10ra*, *Igf2bp1*, *Col4a3* and *Fas*.

### 3.6. Validation of Differential Gene Expression by qRT-PCR

Validation of RNA-seq data was performed by qRT-PCR. The *Mmp2* and *Mmp3* genes were selected for validation based on their level of differential expression and participation in the key pathways enriched in the GO, GSEA and IPA analysis. Consistent with the RNA-seq data, qRT-PCR analysis showed upregulation of both *Mmp2* and *Mmp3* in response to FA which significantly correlated with RNA-seq data using Spearman’s correlation (*Mmp2* R = 0.648, *p* = 0.043; *Mmp3* R = 0.879, *p* = 0.001) (Supplementary Table S13).

### 3.7. FA Intake Induced Differential Expression in Genes that Are Enriched for EZH2 Binding Sites

Chromatin immunoprecipitation sequencing enrichment analysis (ChIP-Seq enrichment analysis) was used to determine whether the differentially expressed genes were enriched as downstream targets for specific transcription factors. ChIP-seq analysis assessed the overlap between differentially expressed genes within the RNA-seq analysis with experimentally validated transcription factor binding sites. This analysis identified 17 differentially expressed genes with EZH2 binding sites within the gene body (Benjamini-Hochberg, *p* = 1.42 × 10^−4^), 19 genes within 500 base pair (bp) upstream or 1000 bp downstream of the transcriptional start site (*p* = 7.30 × 10^−5^), and 10 genes with EZH2 binding sites within both the gene body and 5′ or 3′ regulatory regions; these were *Cadm3*, *col6a2*, *Fam124a*, *Fndc1*, *Lama2*, *Sdk1*, *Tmeff2*, *Tspan11*, *Tubb4a* and *Wnt2* (Supplementary Table S14).

Having identified an enrichment amongst the HFA diet differentially expressed transcripts for EZH2 target genes, we examined the effect of increased FA intake on the expression of a series of polycomb linked epigenetic writers and readers within the RNAseq data set. *Ezh2* expression was nominally downregulated (*p* < 0.05), along with associated PRC2 complex gene *Suz12* and PRC1 complex gene *Bmi1*. Furthermore, *Jarid2*, associated with PRC2 silencing was also nominally downregulated.

## 4. Discussion

Folate plays an essential role in mammary gland development [31,32], however, the relationship between the intake of FA, the synthetic form of folate and BC risk remains complex. To date, human and animal studies have inferred a U-shaped relationship, with both high and low FA intakes associated with increased BC risk. Despite these findings, there are a lack of studies examining the effect of increased FA intake during adulthood on the mammary gland transcriptome, which could highlight important mechanisms by which HFA modulates long-term mammary tumorigenesis. Here, our findings show that increased FA intake during adulthood causes changes to the mammary transcriptome in C57BL/6 mice 4 weeks after the end of the supplementation period. A HFA diet induced changes in genes associated with ECM and EMT and showed enrichment of targets of the key epigenetic regulator of mammary gland morphogenesis, EZH2. These results suggest that a HFA diet can modulate key cellular processes at the transcriptional level which may impact BC susceptibility [33,34,35].

Higher serum levels of 5-methylTHF and FA were found in mice fed a HFA diet, validating the effectiveness of the diet in raising folate and FA status. This was accompanied by increased circulating concentrations of the folate degradation products pAPG and its acetylated metabolite apAPG. Whole body folate status is positively related to folate catabolism [36], thus, one possible explanation for the increase in folate degradation products is that the amount of FA consumed by the HFA mice saturated the whole-body folate pool leading to increased catabolism. If so, this would also be consistent with FA intake exceeding capacity for reduction by dihydrofolate reductase to THF, which is required for assimilation of FA into tissues [37], resulting in circulating unmetabolised FA. An inverse association between unmetabolised FA in plasma and NK cell cytotoxicity has been reported, suggesting free FA may negatively modulate immune function [38], however, the effects on mammary gland function and disease risk are currently unknown.

The present findings show that a HFA diet induces differential changes in the mammary gland transcriptome, with Dynamin 1 (*Dnm1*) the top upregulated gene, and Bcl-2-binding component 3 (*Bbc3)* the top downregulated gene. *Dnm1* plays an essential role in endocytosis and is upregulated in many cancers where it has been suggested to attenuate apoptotic signalling [39], while *Bbc3* which encodes the p53 upregulated modulator of apoptosis (PUMA) is downregulated in some cancers. PUMA is a target of the tumour suppressor protein p53 and is necessary for stress-induced apoptosis [40,41]. PUMA also co-operates with p21 to maintain normal lumen formation in the mammary gland and suppresses EMT [42], with low levels of PUMA in breast carcinomas associated with increased mortality and poorer prognosis [43]. Thus, increased expression of *Dnm1* and decreased expression of PUMA may contribute to breast cancer susceptibility.

HFA intake led to enrichment of the GO terms related to ECM formation and cell migration. In the mammary gland, the ECM provides physical support for the myoepithelial cells and modulates cell fate decisions in mammary progenitor cells [44,45]. Amongst genes related to ECM function that were altered in response to the HFA diet, *Mmp3* expression was significantly upregulated. MMPs are crucial mediators in mammary gland remodelling, with ectopic expression of Mmp3 being reported to induce not only supernumerary branching and eventual tumours [46], but increased production of reactive oxygen species [47] and EMT [48].

Consistent with these findings, the top upregulated hallmark gene sets in the GSEA analysis were linked to EMT and epithelial cell function (apical junction), while the top disease pathway was cancer and metastasis, driven largely by enrichment of EMT genes within this pathway. EMT, which arises through a series of epigenetic alterations, converts polarized and adherent epithelial cells to more motile and invasive mesenchymal-like cells. This transition from epithelial to mesenchymal is a critical process for mammary gland organogenesis [49], while in cancer, EMT facilitates migration, invasion and metastasis. The top upstream regulator predicted amongst the HFA differentially expressed transcripts was alpha catenin, which plays an essential role within adherens junctions [50,51], structures required for maintaining epithelial integrity and which are lost upon EMT. Although the HFA diet induced the upregulation of genes involved in EMT, it did not affect the expression of vimentin or E-cadherin, which are pivotal in this transition process, suggesting HFA intake may increase the expression of remodelling factors at the transcriptional level and enhance the potential of mammary epithelial cells to undergo EMT, rather than promoting EMT itself.

The pathways enriched amongst the downregulated genes were DNA replication (E2F targets), proto-oncogene function (Myc targets), cell cycle progression (G2M checkpoint) and energy metabolism (oxidative phosphorylation). The E2F family of transcription factors which regulate c-Myc expression are involved in regulation of both cell proliferation and apoptosis and can act as both oncogenes and tumour suppressor genes. So, the consequence of a change in E2F expression in relation to HFA intake is unclear. Interestingly, genes involved in oxidative phosphorylation were also downregulated. Some studies have linked a switch from oxidative phosphorylation to glycolysis with EMT [52], while others report a direct correlation between mitochondrial respiration and cancer cell metastasis, with the overexpression of activated receptor gamma coactivator-1 alpha (PGC-1α), which mediates mitochondrial biogenesis [53]. Further metabolic studies will be important to determine whether these changes observed at the level of RNA result in a metabolic switch induced by an HFA diet.

Folate and FA play crucial roles in epigenetic regulation, with Folate/FA-mediated one-carbon metabolism functioning to mediate the supply of methyl groups for DNA, RNA or protein, which modulate transcriptional regulation. HFA intake in rodents has been linked to changes in global DNA methylation and epigenetic regulator expression [16]. Whilst no enrichment of pathways involved in epigenetic gene regulation was shown in the present study, both up- and downregulated genes were enriched for EZH2 target genes. Of note, *Ezh2*, *Suz12* and *Bmi1* were also nominally downregulated within RNA sequencing data. EZH2 is an important epigenetic regulator which has histone methyltransferase activity and, along with Suz12, is a component of the polycomb repressor complex 2 (PRC2), which methylates lysine 27 in histone H3, resulting in gene silencing. EZH2 plays a central role in mammary gland development, as well as in the initiation, progression and metastasis of breast cancer. EZH2 promotes EMT by suppressing expression of epithelial marker E-cadherin (*CDH1*) [54], leading to reduced intercellular adhesion and expression of mesenchymal markers, such as fibronectin, vimentin and N-cadherin. The enrichment of EZH2 target genes amongst the differentially expressed genes suggests that the regulation of these targets may be sensitive to variations in FA intake, which, given the dependence of EZH2 activity on the supply of methyl groups, suggests a mechanism by which FA intake may modulate gene expression profiles. However, enriched targets of EZH2 were both up- and downregulated, and no change in expression was observed of some key EZH2 EMT target genes, suggesting that the changes in the mammary transcriptome represent a complex interplay of many factors, to which EZH2 dysregulation may contribute. Further studies to investigate changes in both histone and DNA methylation in relation to FA intake will be required to assess how the dysregulation of epigenetic processes contributes to the changes in transcription that we observed in this study.

There are some limitations to this study. Firstly, it is not known whether the gene expression changes observed 4 weeks after supplementation occurred during the 4-week HFA supplementation period, or as a result of the switch to the maintenance diet. Nevertheless, this study did show that increased FA intake can induce changes in gene expression that are detectable after the end of the dietary intervention period. We showed that EZH2 targets are enriched amongst the genes affected by HFA intake, suggesting epigenetic processes may be involved, at least in part, in mediating the response to HFA. Secondly, although we saw genome-wide changes in expression, we do not know whether such changes were mirrored by a change in protein expression, and/or a change in mammary gland morphology. Further studies to determine whether the transcriptional changes are accompanied by changes in protein expression and mammary gland morphology will be important to understand the impact that HFA intake has on mammary gland structure and function and the implications on human breast cancer risk. Thirdly, in this study, we only measured gene expression after feeding the control or HFA diet. We did not assess how baseline transcription may change with age, but as the mice were at the same age in both treatment groups our analysis allows the identification of gene expression changes related to the change in diet alone. Nonetheless, it would be interesting to determine whether similar or distinct responses are observed in relation to FA intake during the juvenile period or during pregnancy when there is considerable epigenetic and morphological re-structuring of the mammary gland. Finally, here we have focused on the impact of FA intake on mammary gland gene expression and signalling pathways and have not directly assessed the effect of high FA intake on carcinogenesis. Our data however show that a high FA intake can alter the expression of EMT related genes and oncogenic pathways, but additional studies will be required to assess whether the changes observed in these pathways do contribute directly to an altered susceptibility to mammary tumorigenesis.

Together, the present findings demonstrate that increased FA intake can modify the expression of the mammary gland transcriptome. Thus, one implication of the present findings is that excessive consumption of FA that saturates body folate pools may induce changes in the regulation of the transcription of genes involved in mammary tissue architecture and increase the propensity for EMT, with implications for mammary gland function and tumorigenesis. The results for this study suggest caution with regards to recommendations for net FA intake from multiple sources, particularly in women of reproductive age. Given that breast cancer patients or survivors are likely exposed to increasing levels of FA in the post-fortification era [55], follow-up research is required to further investigate the impact of a high FA intake on the genes and pathways identified in this study and whether high FA induces similar responses in human cells, to fully understand the long-term effects of elevated FA intake on breast cancer susceptibility.

## Figures and Tables

**Table 1:** Plasma folate, folic acid (FA) and folate metabolite concentrations after the end of the 4-week dietary intervention (PND (post-natal day) 102). Values are mean ± SEM for *n* = 8 mice per group. Statistical comparisons were by Student’s unpaired *T*-test. CFA, control folic acid; HFA, high folic acid. Total folate concentration was calculated form the sum of the concentrations of 5-methyltetrahydrofolate (mTHF), 5-formyltetrahydrofolate (5-fTHF), hydroxymethyltetrahydrofolate (hmTHF) and FA. Total folate catabolites were calculated from the sum of para-aminobenzoylglutamnate (pABG) and acetyl para-aminobenzoyl glutamate (acpABG). *n* = 8 females per diet group. Statistical analysis was by Student’s unpaired *T*-test. * *p* < 0.05 and **** *p* < 0.0001.

Metabolite (nmol/L)	Dietary Group		
	CFA	HFA	*p*
Plasma 5 mTHF	47.26 ± 3.16	76.79 ± 3.49	<0.0001 ****
Plasma 5 fTHF	0.84 ± 0.55	1.30 ± 0.63	0.59
Folic Acid	0.31 ± 0.31	1.80 ± 0.43	0.01*
hmTHF	2.02 ± 0.42	2.16 ± 0.55	0.85
Total folates	50.45 ± 3.57	82.05 ± 4.47	<0.0001 ****
pABG	34.09 ± 2.8	53.45 ± 8.26	0.04 *
apABG	3.46 ± 0.28	4.99 ± 0.74	0.07
Total folate catabolites	37.54 ± 3.07	58.44 ± 8.90	0.04 *

**Table 2:** Top 10 genes upregulated (+logFC) or downregulated (−logFC) by HFA intake.

Mgi Ref	LogFC	FDR	*p* Value	Gene Name
*Upregulated genes*				
*Dnm1*	1.962515	9.71 × 10^−6^	7.91 × 10^−10^	dynamin 1
*Cd209g*	2.787528	9.80 × 10^−6^	1.60 × 10^−9^	CD209g antigen
*Gabra3*	3.202282	1.24 × 10^−5^	4.03 × 10^−9^	GABA A receptor, subunit alpha 3
*Tubb4a*	1.970152	1.24 × 10^−5^	3.92 × 10^−9^	tubulin, beta 4A class IVA
*Col6a2*	1.980787	2.06 × 10^−5^	1.01 × 10^−8^	collagen, type VI, alpha 2
*Ackr3*	1.898499	2.06 × 10^−5^	8.57 × 10^−9^	atypical chemokine receptor 3
*Cd209f*	3.04923	2.24 × 10^−5^	1.28 × 10^−8^	CD209f antigen
*C5ar1*	2.681699	2.90 × 10^−5^	1.89 × 10^−8^	complement component 5a receptor 1
*Col6a3*	2.228025	5.76 × 10^−5^	4.22 × 10^−8^	collagen, type VI, alpha 3
*Itga11*	3.179464	7.97 × 10^−5^	6.50 × 10^−8^	integrin alpha 11
*Downregulated genes*				
*Bbc3*	−1.76456	0.000166	1.88 × 10^−7^	BCL2 binding component 3
*Snhg12*	−1.39266	0.000811	1.85 × 10^−6^	small nucleolar RNA host gene 12
*Rpl22l1*	−1.74953	0.000885	2.09 × 10^−6^	ribosomal protein L22 like 1
*Tbca*	−0.97069	0.002674	1.09 × 10^−5^	tubulin cofactor A
*mir-703*	−1.69341	0.003125	1.40 × 10^−5^	mmu-mir-703
*Mphosph6*	−1.19544	0.003226	1.50 × 10^−5^	M phase phosphoprotein 6
*Itpr2*	−0.90467	0.004558	2.24 × 10^−5^	inositol 1,4,5-triphosphate receptor 2
*Impdh1*	−0.98072	0.005139	3.06 × 10^−5^	inosine 5’-phosphate dehydrogenase 1
*Tpt1*	−1.20074	0.010028	8.66 × 10^−5^	tumour protein, translationally controlled 1
*Tdp2*	−0.8294	0.012004	0.000118	tyrosyl-DNA phosphodiesterase 2

FC = fold change; FDR = false discovery rate; HFA: high FA; GABA: Gamma-Aminobutyric Acid; IVA: Individual voluntary arrangement; BCL 2: B-cell lymphoma-2.

## Supplementary Materials

The following are available online at https://www.mdpi.com/2072-6643/12/9/2821/s1, Figure S1: MA plot and Volcano plot. Figure S2 (A) Top 3 disease pathways; (B) Mammary tumour disease pathway (C) Top upstream regulator pathway. Table S1: Dietary compositions. Table S2: qRT-PCR primer information. Table S3: Growth and energy intakes. Table S4: Differentially expressed genes between CFA and HFA groups. Table S5: Gene Ontology. Table S6: GSEA analysis (Hallmark). Table S7: GSEA analysis (Hallmark EMT). Table S8: GSEA analysis (Hallmark E2F targets). Table S9: GSEA analysis (C2). Table S10: Top disease pathways. Table S11: Top 5 networks Table S12: Top upstream regulators identified from Ingenuity Pathway Analysis. Table S13: Chip-seq enrichment analysis. Table S14: Validation of differentially expressed Genes within RNA-seq data.
