# Supplementary material for: Folic Acid Induces Intake-Related Changes in the Mammary Tissue Transcriptome of C57BL/6 Mice
Source: Nutrients. 2020 Sep 15;12(9):2821. doi: 10.3390/nu12092821 (PMC7551343; doi:10.3390/nu12092821)
Supplement: Supplementary file 1 [file nutrients-12-02821-s001.pdf]

Supplementary data

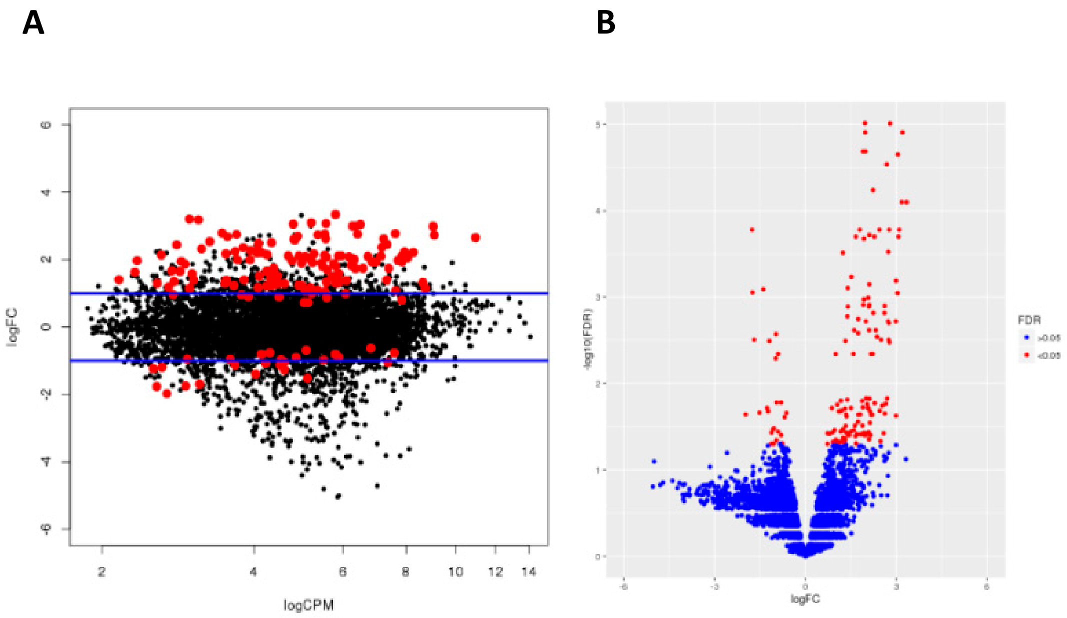

Supplementary Figure 1: MA plot and Volcano plot from mice supplemented with CFA or HFA. **A** MA plot showing average logCPM against logFC (change in log2 CPM). Individual genes are represented by single dots. Differentially expressed genes (FDR < 0.05) appear in red. **B** Volcano plot of logFC against – logFDR (-log10 of the FDR). Genes that demonstrate significant differential expression are shown in red (FDR < 0.05) (n = 5 per group).

Supplementary data

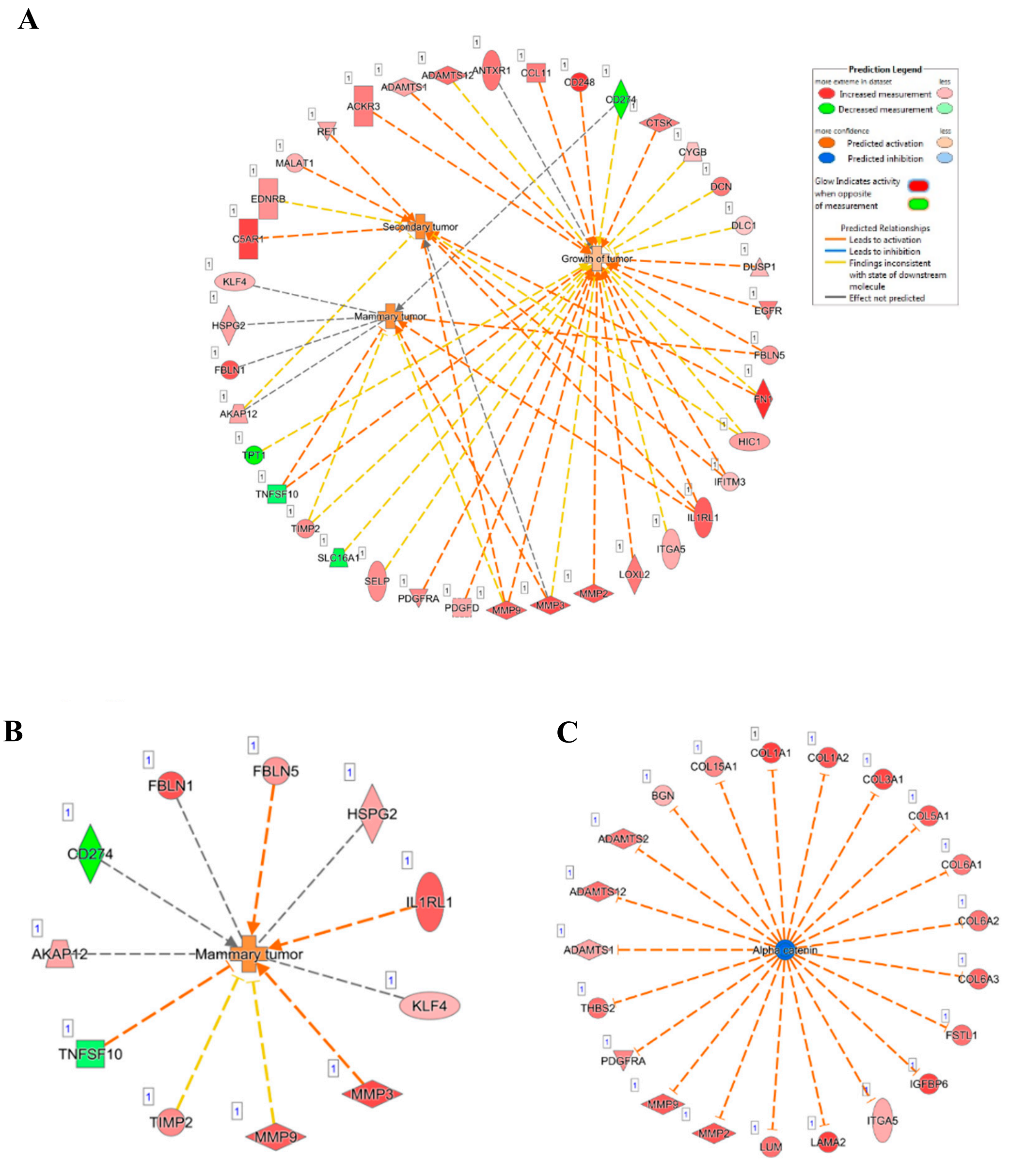

Supplementary Figure 2: **A** Top 3 disease pathways; **B** Mammary tumour disease pathway; **C** Top upstream regulator pathway.

Supplementary data

Supplementary Table 1: Dietary compositions.

**Nutritional Profile**	**CFA (mg/kg)**	**HFA (mg/kg)**	**AIN-93M (mg/kg)**
Folic Acid	1.000	5.100	2.100
Vitamin B_{12}	0.027	0.027	0.028
Corn Starch	465.1	465.1	465.6
Maltodextrin	155.0	155.0	155.0
Casein	140.0	140.0	140.0
Sucrose	100.0	100.0	100.0
Powdered Cellulose	50.00	50.00	50.00
Soybean Oil	40.00	40.00	40.00
AIN 93M Mineral Mix**	35.00	35.00	35.00
AIN 93M Vitamin Mix*	10.00	10.00	10.00
Choline Bitartrate	2.500	2.500	2.500
L-Cystine	1.800	1.800	1.800
t-Butylhydroquinone	0.008	0.008	0.008
**Nutritional Profile**	%	%	%
Fat	4.1	4.1	4.1
Protein	13.0	13.0	13.0
Carbohydrate	73	72.1	73.0
Total Energy (kcal/g)	3.81	3.77	3.81

* Vitamin Mix: Vitamin A, 4 IU/kg; Vitamin D-3, 1 IU/kg; Vitamin E, 78.8 IU/kg; Vitamin K, 0.75 mg/kg; Thiamine Hydrochloride, 6.0 mg/kg; Riboflavin, 6.5 mg/kg; Niacin, 30 mg/kg; Pantothenic Acid, 16 mg/kg; Pyridoxine, 5.8 mg/kg; Biotin, 0.2 mg/kg; Choline, 1080.14 mg/kg; Ascorbic Acid, 2.59 mg/kg. ** Minerals: Calcium, 0.50-0.51 %; Phosphorus, 0.31 %; Potassium, 0.36 %; Magnesium, 0.05 %; Sodium, 0.13 %; Chloride, 0.20 %; Fluorine, 1.0 mg/kg; Iron, 39 mg/kg; Zinc, 35 mg/kg; Manganese, 11 mg/kg; Copper, 6.0 mg/kg; Cobalt, 0.0 mg/kg; Iodine, 0.21, mg/kg; Chromium, 1.0 mg/kg; Molybdenum, 0.14 mg/kg; Selenium, 0.22 mg/kg.

Supplementary data

Supplementary Table 2: qRT-PCR primer information.

Gene	Forward Primer (5′-3′)<br>Reverse Primer (3′-5′)	Reference	Product Size (bp)	Annealing Temp (◦C)
Mmp2	Quantitect Proprietary Sequence (Qiagen, UK)	QT00116116	145	60
Mmp3	Quantitect Proprietary Sequence (Qiagen, UK)	QT00107751	146	60

Supplementary data

Supplementary Table 3: Growth and energy intakes.

**Growth and energy intake**	**Dietary Group**		**Significance (T test)**
**PND 74-102**	**CFA**	**HFA**	**p**
Body Weight	492.1 ± 5.27 *	493.2 ± 8.58 *	0.91
Energy Intake	491.6 ± 7.50 *	489.9 ± 11.63 *	0.89
**PND 102-130**			
Body Weight	492.1 ± 5.27 *	493.2 ± 8.58 *	0.91
Energy Intake	491.6 ± 7.50 *	489.9 ± 11.63 *	0.89

*AUC arbitrary units

Supplementary data

Supplementary Table 4: Differentially expressed genes between CFA and HFA groups.

Gene	logFC	PValue	FDR	mgi_symbol	description
ENSMUSG00000026825	1.962515	7.91E-10	9.71E-06	Dnm1	dynamin 1 [Source:MGI Symbol;Acc:MGI:107384]
ENSMUSG00000079168	2.787528	1.60E-09	9.80E-06	Cd209g	CD209g antigen [Source:MGI Symbol;Acc:MGI:1917442]
ENSMUSG00000031343	3.202282	4.03E-09	1.24E-05	Gabra3	gamma-aminobutyric acid (GABA) A receptor, subunit alpha 3 [Source:MGI Symbol;Acc:MGI:95615]
ENSMUSG00000062591	1.970152	3.92E-09	1.24E-05	Tubb4a	tubulin, beta 4A class IVA [Source:MGI Symbol;Acc:MGI:107848]
ENSMUSG00000020241	1.980787	1.01E-08	2.06E-05	Col6a2	collagen, type VI, alpha 2 [Source:MGI Symbol;Acc:MGI:88460]
ENSMUSG00000044337	1.898499	8.57E-09	2.06E-05	Ackr3	atypical chemokine receptor 3 [Source:MGI Symbol;Acc:MGI:109562]
ENSMUSG00000051906	3.04923	1.28E-08	2.24E-05	Cd209f	CD209f antigen [Source:MGI Symbol;Acc:MGI:1916392]
ENSMUSG00000049130	2.681699	1.89E-08	2.90E-05	C5ar1	complement component 5a receptor 1 [Source:MGI Symbol;Acc:MGI:88232]
ENSMUSG00000048126	2.228025	4.22E-08	5.76E-05	Col6a3	collagen, type VI, alpha 3 [Source:MGI Symbol;Acc:MGI:88461]
ENSMUSG00000032243	3.179464	6.50E-08	7.97E-05	Itga11	integrin alpha 11 [Source:MGI Symbol;Acc:MGI:2442114]
ENSMUSG00000056481	3.33967	7.16E-08	7.99E-05	Cd248	CD248 antigen, endosialin [Source:MGI Symbol;Acc:MGI:1917695]
ENSMUSG00000044017	3.095523	2.16E-07	0.000166	Adgrd1	adhesion G protein-coupled receptor D1 [Source:MGI Symbol;Acc:MGI:3041203]
ENSMUSG00000019122	2.761874	1.69E-07	0.000166	Ccl9	chemokine (C-C motif) ligand 9 [Source:MGI Symbol;Acc:MGI:104533]
ENSMUSG00000029925	2.437321	2.15E-07	0.000166	Tbxas1	thromboxane A synthase 1, platelet [Source:MGI Symbol;Acc:MGI:98497]
ENSMUSG00000047793	1.79296	2.09E-07	0.000166	Sned1	sushi, nidogen and EGF-like domains 1 [Source:MGI Symbol;Acc:MGI:3045960]
ENSMUSG00000002083	-1.76456	1.88E-07	0.000166	Bbc3	BCL2 binding component 3 [Source:MGI Symbol;Acc:MGI:2181667]
ENSMUSG00000020695	2.106666	2.64E-07	0.000191	Mrc2	mannose receptor, C type 2 [Source:MGI Symbol;Acc:MGI:107818]
ENSMUSG00000008845	3.070336	3.18E-07	0.0002	Cd163	CD163 antigen [Source:MGI Symbol;Acc:MGI:2135946]
ENSMUSG00000029373	2.273035	2.94E-07	0.0002	Pf4	platelet factor 4 [Source:MGI Symbol;Acc:MGI:1888711]
ENSMUSG00000005338	1.659054	3.26E-07	0.0002	Cadm3	cell adhesion molecule 3 [Source:MGI Symbol;Acc:MGI:2137858]
ENSMUSG00000001119	1.929115	3.60E-07	0.00021	Col6a1	collagen, type VI, alpha 1 [Source:MGI Symbol;Acc:MGI:88459]

ENSMUSG00000024053	2.732957	5.37E-07	0.000299	Emilin2	elastin microfibril interfacer 2 [Source:MGI Symbol;Acc:MGI:2389136]
ENSMUSG00000000555	1.230958	5.74E-07	0.000307	Itga5	integrin alpha 5 (fibronectin receptor alpha) [Source:MGI Symbol;Acc:MGI:96604]
ENSMUSG00000032186	1.512622	1.14E-06	0.000584	Tmod2	tropomodulin 2 [Source:MGI Symbol;Acc:MGI:1355335]
ENSMUSG00000018927	2.989764	1.32E-06	0.000649	Ccl6	chemokine (C-C motif) ligand 6 [Source:MGI Symbol;Acc:MGI:98263]
ENSMUSG00000028108	2.105854	1.51E-06	0.000711	Ecm1	extracellular matrix protein 1 [Source:MGI Symbol;Acc:MGI:103060]
ENSMUSG00000035184	1.394884	1.72E-06	0.000784	Fam124a	family with sequence similarity 124, member A [Source:MGI Symbol;Acc:MGI:3645930]
ENSMUSG00000086290	-1.39266	1.85E-06	0.000811	Snhg12	small nucleolar RNA host gene 12 [Source:MGI Symbol;Acc:MGI:1916721]
ENSMUSG00000039221	-1.74953	2.09E-06	0.000885	Rpl22l1	ribosomal protein L22 like 1 [Source:MGI Symbol;Acc:MGI:1915278]
ENSMUSG00000039109	3.039735	2.20E-06	0.0009	F13a1	coagulation factor XIII, A1 subunit [Source:MGI Symbol;Acc:MGI:1921395]
ENSMUSG00000037206	2.068572	2.57E-06	0.001019	Islr	immunoglobulin superfamily containing leucine-rich repeat [Source:MGI Symbol;Acc:MGI:1349645]
ENSMUSG00000016494	1.926517	2.78E-06	0.001065	Cd34	CD34 antigen [Source:MGI Symbol;Acc:MGI:88329]
ENSMUSG00000020681	2.10306	3.41E-06	0.001231	Ace	angiotensin I converting enzyme (peptidyl-dipeptidase A) 1 [Source:MGI Symbol;Acc:MGI:87874]
ENSMUSG00000020676	1.908838	3.40E-06	0.001231	Ccl11	chemokine (C-C motif) ligand 11 [Source:MGI Symbol;Acc:MGI:103576]
ENSMUSG00000033327	2.614372	3.61E-06	0.001265	Tnxb	tenascin XB [Source:MGI Symbol;Acc:MGI:1932137]
ENSMUSG00000026674	2.093032	3.88E-06	0.001286	Ddr2	discoidin domain receptor family, member 2 [Source:MGI Symbol;Acc:MGI:1345277]
ENSMUSG00000063160	1.399783	3.82E-06	0.001286	Numbl	numb-like [Source:MGI Symbol;Acc:MGI:894702]
ENSMUSG00000040950	2.605377	4.85E-06	0.001526	Mgl2	macrophage galactose N-acetyl-galactosamine specific lectin 2 [Source:MGI Symbol;Acc:MGI:2385729]
ENSMUSG00000020312	2.236769	4.79E-06	0.001526	Shc2	SHC (Src homology 2 domain containing) transforming protein 2 [Source:MGI Symbol;Acc:MGI:106180]
ENSMUSG00000029718	1.380253	5.44E-06	0.00167	Pcolce	procollagen C-endopeptidase enhancer protein [Source:MGI Symbol;Acc:MGI:105099]
ENSMUSG00000029231	1.738631	5.98E-06	0.001791	Pdgfra	platelet derived growth factor receptor, alpha polypeptide [Source:MGI Symbol;Acc:MGI:97530]
ENSMUSG00000026193	2.981304	6.86E-06	0.001912	Fn1	fibronectin 1 [Source:MGI Symbol;Acc:MGI:95566]
ENSMUSG00000001506	2.726791	6.77E-06	0.001912	Col1a1	collagen, type I, alpha 1 [Source:MGI Symbol;Acc:MGI:88467]
ENSMUSG00000029659	1.992774	6.79E-06	0.001912	Medag	mesenteric estrogen dependent adipogenesis [Source:MGI Symbol;Acc:MGI:1917967]
ENSMUSG00000019899	2.75248	7.32E-06	0.001996	Lama2	laminin, alpha 2 [Source:MGI Symbol;Acc:MGI:99912]
ENSMUSG00000026069	2.317838	9.45E-06	0.002416	Il1rl1	interleukin 1 receptor-like 1 [Source:MGI Symbol;Acc:MGI:98427]

ENSMUSG00000000753	2.098949	9.34E-06	0.002416	Serpinf1	serine (or cysteine) peptidase inhibitor, clade F, member 1 [Source:MGI Symbol;Acc:MGI:108080]
ENSMUSG00000035107	1.604302	9.06E-06	0.002416	Dcbld2	discoidin, CUB and LCCL domain containing 2 [Source:MGI Symbol;Acc:MGI:1920629]
ENSMUSG00000028517	1.737354	1.04E-05	0.002611	Ppap2b	phosphatidic acid phosphatase type 2B [Source:MGI Symbol;Acc:MGI:1915166]
ENSMUSG00000042043	-0.97069	1.09E-05	0.002674	Tbca	tubulin cofactor A [Source:MGI Symbol;Acc:MGI:107549]
ENSMUSG00000026837	2.366063	1.22E-05	0.002881	Col5a1	collagen, type V, alpha 1 [Source:MGI Symbol;Acc:MGI:88457]
ENSMUSG00000017737	2.355823	1.21E-05	0.002881	Mmp9	matrix metallopeptidase 9 [Source:MGI Symbol;Acc:MGI:97011]
ENSMUSG00000044749	2.746114	1.36E-05	0.003125	Abca6	ATP-binding cassette, sub-family A (ABC1), member 6 [Source:MGI Symbol;Acc:MGI:1923434]
ENSMUSG00000071656	2.499824	1.38E-05	0.003125	Lrrn4cl	LRRN4 C-terminal like [Source:MGI Symbol;Acc:MGI:1916102]
ENSMUSG00000105814	-1.69341	1.40E-05	0.003125		mmu-mir-703 [Source:miRBase;Acc:MI0004687]
ENSMUSG00000038587	1.31517	1.48E-05	0.003226	Akap12	A kinase (PRKA) anchor protein (gravin) 12 [Source:MGI Symbol;Acc:MGI:1932576]
ENSMUSG00000031843	-1.19544	1.50E-05	0.003226	Mphosph6	M phase phosphoprotein 6 [Source:MGI Symbol;Acc:MGI:1915783]
ENSMUSG00000061100	2.769118	1.58E-05	0.003343	Retnla	resistin like alpha [Source:MGI Symbol;Acc:MGI:1888504]
ENSMUSG00000026574	2.214016	2.26E-05	0.004558	Dpt	dermatopontin [Source:MGI Symbol;Acc:MGI:1928392]
ENSMUSG00000017446	2.165937	2.30E-05	0.004558	C1qtnf1	C1q and tumor necrosis factor related protein 1 [Source:MGI Symbol;Acc:MGI:1919254]
ENSMUSG00000027254	1.579555	2.29E-05	0.004558	Map1a	microtubule-associated protein 1 A [Source:MGI Symbol;Acc:MGI:1306776]
ENSMUSG00000030287	-0.90467	2.24E-05	0.004558	Itpr2	inositol 1,4,5-triphosphate receptor 2 [Source:MGI Symbol;Acc:MGI:99418]
ENSMUSG00000009418	0.992554	2.35E-05	0.004569	Nav1	neuron navigator 1 [Source:MGI Symbol;Acc:MGI:2183683]
ENSMUSG00000031906	3.752212	2.91E-05	0.005139	Smpd3	sphingomyelin phosphodiesterase 3, neutral [Source:MGI Symbol;Acc:MGI:1927578]
ENSMUSG00000022371	2.425037	2.94E-05	0.005139	Col14a1	collagen, type XIV, alpha 1 [Source:MGI Symbol;Acc:MGI:1341272]
ENSMUSG00000032334	1.978786	2.83E-05	0.005139	Loxl1	lysyl oxidase-like 1 [Source:MGI Symbol;Acc:MGI:106096]
ENSMUSG00000034205	1.961196	3.03E-05	0.005139	Loxl2	lysyl oxidase-like 2 [Source:MGI Symbol;Acc:MGI:2137913]
ENSMUSG00000020122	1.884449	3.02E-05	0.005139	Egfr	epidermal growth factor receptor [Source:MGI Symbol;Acc:MGI:95294]
ENSMUSG00000022122	1.577547	3.01E-05	0.005139	Ednrb	endothelin receptor type B [Source:MGI Symbol;Acc:MGI:102720]
ENSMUSG00000031494	1.510783	2.88E-05	0.005139	Cd209a	CD209a antigen [Source:MGI Symbol;Acc:MGI:2157942]
ENSMUSG00000043943	1.449324	2.78E-05	0.005139	Naalad2	N-acetylated alpha-linked acidic dipeptidase 2 [Source:MGI Symbol;Acc:MGI:1919810]
ENSMUSG00000022180	1.255186	3.04E-05	0.005139	Slc7a8	solute carrier family 7, member 8 [Source:MGI Symbol;Acc:MGI:1355323]
ENSMUSG00000003500	-0.98072	3.06E-05	0.005139	Impdh1	inosine 5'-phosphate dehydrogenase 1 [Source:MGI Symbol;Acc:MGI:96567]
ENSMUSG00000026255	2.301714	3.23E-05	0.005293	Efhd1	EF hand domain containing 1 [Source:MGI Symbol;Acc:MGI:1921607]

ENSMUSG00000026315	1.23721	3.21E-05	0.005293	Serpinb8	serine (or cysteine) peptidase inhibitor, clade B, member 8 [Source:MGI Symbol;Acc:MGI:894657]
ENSMUSG00000017466	1.689254	3.46E-05	0.005511	Timp2	tissue inhibitor of metalloproteinase 2 [Source:MGI Symbol;Acc:MGI:98753]
ENSMUSG00000028996	1.389736	3.46E-05	0.005511	Rbp7	retinol binding protein 7, cellular [Source:MGI Symbol;Acc:MGI:1890409]
ENSMUSG00000097451	1.880883	3.65E-05	0.00574	Rian	RNA imprinted and accumulated in nucleus [Source:MGI Symbol;Acc:MGI:1922995]
ENSMUSG00000021876	2.113216	3.75E-05	0.005822	Rnase4	ribonuclease, RNase A family 4 [Source:MGI Symbol;Acc:MGI:1926217]
ENSMUSG00000036412	2.940951	3.89E-05	0.005942	Arsi	arylsulfatase i [Source:MGI Symbol;Acc:MGI:2670959]
ENSMUSG00000039883	2.508339	3.92E-05	0.005942	Lrrc17	leucine rich repeat containing 17 [Source:MGI Symbol;Acc:MGI:1921761]
ENSMUSG00000024084	1.892018	4.39E-05	0.006568	Qpct	glutaminyl-peptide cyclotransferase (glutaminyl cyclase) [Source:MGI Symbol;Acc:MGI:1917786]
ENSMUSG00000027377	1.345486	4.77E-05	0.007054	Mall	mal, T cell differentiation protein-like [Source:MGI Symbol;Acc:MGI:2385152]
ENSMUSG00000038463	2.41534	4.84E-05	0.007069	Olfml2b	olfactomedin-like 2B [Source:MGI Symbol;Acc:MGI:2443310]
ENSMUSG00000052821	1.462333	4.92E-05	0.0071	Cysltr1	cysteinyl leukotriene receptor 1 [Source:MGI Symbol;Acc:MGI:1926218]
ENSMUSG00000029661	2.460243	5.26E-05	0.0075	Col1a2	collagen, type I, alpha 2 [Source:MGI Symbol;Acc:MGI:88468]
ENSMUSG00000017969	2.31129	5.32E-05	0.007507	Ptgis	prostaglandin I2 (prostacyclin) synthase [Source:MGI Symbol;Acc:MGI:1097156]
ENSMUSG00000005397	1.681812	5.46E-05	0.007614	Nid1	nidogen 1 [Source:MGI Symbol;Acc:MGI:97342]
ENSMUSG00000021390	2.524996	5.57E-05	0.007683	Ogn	osteoglycin [Source:MGI Symbol;Acc:MGI:109278]
ENSMUSG00000028339	1.736581	5.75E-05	0.007846	Col15a1	collagen, type XV, alpha 1 [Source:MGI Symbol;Acc:MGI:88449]
ENSMUSG00000070942	1.741307	5.91E-05	0.007965	Il1rl2	interleukin 1 receptor-like 2 [Source:MGI Symbol;Acc:MGI:1913107]
ENSMUSG00000043099	1.376376	6.09E-05	0.008121	Hic1	hypermethylated in cancer 1 [Source:MGI Symbol;Acc:MGI:1338010]
ENSMUSG00000035279	2.054824	6.25E-05	0.00825	Ssc5d	scavenger receptor cysteine rich family, 5 domains [Source:MGI Symbol;Acc:MGI:3606211]
ENSMUSG00000018906	1.102495	6.37E-05	0.008311	P4ha2	procollagen-proline, 2-oxoglutarate 4-dioxygenase, alpha II polypeptide [Source:MGI Symbol;Acc:MGI:894286]
ENSMUSG00000023885	2.080238	6.94E-05	0.008803	Thbs2	thrombospondin 2 [Source:MGI Symbol;Acc:MGI:98738]
ENSMUSG00000054580	1.352433	6.93E-05	0.008803	Pla2r1	phospholipase A2 receptor 1 [Source:MGI Symbol;Acc:MGI:102468]
ENSMUSG00000023411	1.041771	6.96E-05	0.008803	Nfatc4	nuclear factor of activated T cells, cytoplasmic, calcineurin dependent 4 [Source:MGI Symbol;Acc:MGI:1920431]
ENSMUSG00000039683	2.316999	7.38E-05	0.009184	Sdk1	sidekick homolog 1 (chicken) [Source:MGI Symbol;Acc:MGI:2444413]
ENSMUSG00000040310	2.305193	7.41E-05	0.009184	Alx4	aristaless-like homeobox 4 [Source:MGI Symbol;Acc:MGI:108359]
ENSMUSG00000024011	3.698289	7.72E-05	0.009287	Pi16	peptidase inhibitor 16 [Source:MGI Symbol;Acc:MGI:1921366]

ENSMUSG00000034463	1.918364	7.65E-05	0.009287	Scara3	scavenger receptor class A, member 3 [Source:MGI Symbol;Acc:MGI:2444418]
ENSMUSG00000020044	1.456522	7.66E-05	0.009287	Timp3	tissue inhibitor of metalloproteinase 3 [Source:MGI Symbol;Acc:MGI:98754]
ENSMUSG00000039529	1.396946	8.25E-05	0.009834	Atp8b1	ATPase, class I, type 8B, member 1 [Source:MGI Symbol;Acc:MGI:1859665]
ENSMUSG00000026840	1.592717	8.34E-05	0.009836	Lamc3	laminin gamma 3 [Source:MGI Symbol;Acc:MGI:1344394]
ENSMUSG00000041797	2.04293	8.57E-05	0.010012	Abca9	ATP-binding cassette, sub-family A (ABC1), member 9 [Source:MGI Symbol;Acc:MGI:2386796]
ENSMUSG00000060126	-1.20074	8.66E-05	0.010028	Tpt1	tumor protein, translationally-controlled 1 [Source:MGI Symbol;Acc:MGI:104890]
ENSMUSG00000041120	1.976255	8.85E-05	0.01015	Nbl1	neuroblastoma, suppression of tumorigenicity 1 [Source:MGI Symbol;Acc:MGI:104591]
ENSMUSG00000054203	2.172726	9.48E-05	0.010253	Ifi205	interferon activated gene 205 [Source:MGI Symbol;Acc:MGI:101847]
ENSMUSG00000019929	1.976795	9.36E-05	0.010253	Dcn	decorin [Source:MGI Symbol;Acc:MGI:94872]
ENSMUSG00000031963	1.774257	9.44E-05	0.010253	Bmper	BMP-binding endothelial regulator [Source:MGI Symbol;Acc:MGI:1920480]
ENSMUSG00000045934	1.52195	9.19E-05	0.010253	Mtmr11	myotubularin related protein 11 [Source:MGI Symbol;Acc:MGI:2652817]
ENSMUSG00000021186	1.509179	9.33E-05	0.010253	Fbln5	fibulin 5 [Source:MGI Symbol;Acc:MGI:1346091]
ENSMUSG00000029122	1.221093	9.52E-05	0.010253	Evc	Ellis van Creveld gene syndrome [Source:MGI Symbol;Acc:MGI:1890596]
ENSMUSG00000025492	0.932351	9.42E-05	0.010253	Ifitm3	interferon induced transmembrane protein 3 [Source:MGI Symbol;Acc:MGI:1913391]
ENSMUSG00000023046	2.704725	0.000104	0.011127	Igfbp6	insulin-like growth factor binding protein 6 [Source:MGI Symbol;Acc:MGI:96441]
ENSMUSG00000004098	2.068609	0.000106	0.011218	Col5a3	collagen, type V, alpha 3 [Source:MGI Symbol;Acc:MGI:1858212]
ENSMUSG00000002847	2.687844	0.000109	0.011374	Pla1a	phospholipase A1 member A [Source:MGI Symbol;Acc:MGI:1934677]
ENSMUSG00000025784	2.159297	0.000109	0.011374	Clec3b	C-type lectin domain family 3, member b [Source:MGI Symbol;Acc:MGI:104540]
ENSMUSG00000021702	2.154367	0.000115	0.01183	Thbs4	thrombospondin 4 [Source:MGI Symbol;Acc:MGI:1101779]
ENSMUSG00000058624	1.219928	0.000119	0.012004	Gda	guanine deaminase [Source:MGI Symbol;Acc:MGI:95678]
ENSMUSG00000019948	-0.80862	0.000119	0.012004	Actr6	ARP6 actin-related protein 6 [Source:MGI Symbol;Acc:MGI:1914269]
ENSMUSG00000035958	-0.8294	0.000118	0.012004	Tdp2	tyrosyl-DNA phosphodiesterase 2 [Source:MGI Symbol;Acc:MGI:1860486]
ENSMUSG00000066113	2.492664	0.000123	0.012233	Adamtsl1	ADAMTS-like 1 [Source:MGI Symbol;Acc:MGI:1924989]
ENSMUSG00000027204	2.782546	0.000147	0.014469	Fbn1	fibrillin 1 [Source:MGI Symbol;Acc:MGI:95489]
ENSMUSG00000026064	-0.805	0.000147	0.014469	Ptp4a1	protein tyrosine phosphatase 4a1 [Source:MGI Symbol;Acc:MGI:1277096]
ENSMUSG00000021943	1.432369	0.00015	0.014503	Gdf10	growth differentiation factor 10 [Source:MGI Symbol;Acc:MGI:95684]
ENSMUSG00000039480	-0.85606	0.00015	0.014503	Nt5dc1	5'-nucleotidase domain containing 1 [Source:MGI Symbol;Acc:MGI:2442446]
ENSMUSG00000033420	2.001399	0.000154	0.014807	Antxr1	anthrax toxin receptor 1 [Source:MGI Symbol;Acc:MGI:1916788]
ENSMUSG00000037379	2.69445	0.000156	0.014871	Spon2	spondin 2, extracellular matrix protein [Source:MGI Symbol;Acc:MGI:1923724]

ENSMUSG00000015852	2.110559	0.000159	0.014964	Fcrls	Fc receptor-like S, scavenger receptor [Source:MGI Symbol;Acc:MGI:1933397]
ENSMUSG00000017754	1.379215	0.000164	0.015399	Pltp	phospholipid transfer protein [Source:MGI Symbol;Acc:MGI:103151]
ENSMUSG00000030865	1.16902	0.00017	0.015833	Chp2	calcineurin-like EF hand protein 2 [Source:MGI Symbol;Acc:MGI:1917511]
ENSMUSG00000051748	1.868341	0.000173	0.015954	Wfdc21	WAP four-disulfide core domain 21 [Source:MGI Symbol;Acc:MGI:1913357]
ENSMUSG00000032902	-0.80707	0.000183	0.016642	Slc16a1	solute carrier family 16 (monocarboxylic acid transporters), member 1 [Source:MGI Symbol;Acc:MGI:106013]
ENSMUSG00000051224	-0.95393	0.000182	0.016642	Tceanc	transcription elongation factor A (SII) N-terminal and central domain containing [Source:MGI Symbol;Acc:MGI:2685236]
ENSMUSG00000028047	2.282829	0.000187	0.016913	Thbs3	thrombospondin 3 [Source:MGI Symbol;Acc:MGI:98739]
ENSMUSG00000026043	2.651278	0.000192	0.01716	Col3a1	collagen, type III, alpha 1 [Source:MGI Symbol;Acc:MGI:88453]
ENSMUSG00000036545	2.034725	0.000195	0.017319	Adamts2	a disintegrin-like and metallopeptidase (reprolysin type) with thrombospondin type 1 motif, 2 [Source:MGI Symbol;Acc:MGI:1347356]
ENSMUSG00000003032	1.04926	0.000199	0.017613	Klf4	Kruppel-like factor 4 (gut) [Source:MGI Symbol;Acc:MGI:1342287]
ENSMUSG00000026938	2.535281	0.000206	0.018096	Fcna	ficolin A [Source:MGI Symbol;Acc:MGI:1340905]
ENSMUSG00000028111	1.890661	0.000217	0.018899	Ctsk	cathepsin K [Source:MGI Symbol;Acc:MGI:107823]
ENSMUSG00000037060	1.497985	0.000223	0.019232	Prkcdbp	protein kinase C, delta binding protein [Source:MGI Symbol;Acc:MGI:1923422]
ENSMUSG00000056427	2.075112	0.000229	0.019244	Slit3	slit homolog 3 (Drosophila) [Source:MGI Symbol;Acc:MGI:1315202]
ENSMUSG00000022816	2.054723	0.000228	0.019244	Fstl1	follistatin-like 1 [Source:MGI Symbol;Acc:MGI:102793]
ENSMUSG00000020810	0.8674	0.000229	0.019244	Cygb	cytoglobin [Source:MGI Symbol;Acc:MGI:2149481]
ENSMUSG00000016496	-1.26747	0.000229	0.019244	Cd274	CD274 antigen [Source:MGI Symbol;Acc:MGI:1926446]
ENSMUSG00000022656	1.304421	0.000235	0.019606	Pvrl3	poliovirus receptor-related 3 [Source:MGI Symbol;Acc:MGI:1930171]
ENSMUSG00000084960	1.186977	0.000248	0.020597	B430010I23Rik	RIKEN cDNA B430010I23 gene [Source:MGI Symbol;Acc:MGI:1926099]
ENSMUSG00000031740	2.447916	0.000251	0.020706	Mmp2	matrix metallopeptidase 2 [Source:MGI Symbol;Acc:MGI:97009]
ENSMUSG00000092341	1.153621	0.000256	0.020949	Malat1	metastasis associated lung adenocarcinoma transcript 1 (non-coding RNA) [Source:MGI Symbol;Acc:MGI:1919539]
ENSMUSG00000026389	1.095449	0.00026	0.020966	Steap3	STEAP family member 3 [Source:MGI Symbol;Acc:MGI:1915678]
ENSMUSG00000022948	-1.23327	0.000258	0.020966	Setd4	SET domain containing 4 [Source:MGI Symbol;Acc:MGI:2136890]
ENSMUSG00000004814	2.13443	0.000263	0.021094	Ccl24	chemokine (C-C motif) ligand 24 [Source:MGI Symbol;Acc:MGI:1928953]
ENSMUSG00000052192	-1.51961	0.000272	0.021709	Gm5963	predicted pseudogene 5963 [Source:MGI Symbol;Acc:MGI:3648418]
ENSMUSG00000026434	-0.62683	0.000276	0.021851	Nucks1	nuclear casein kinase and cyclin-dependent kinase substrate 1 [Source:MGI Symbol;Acc:MGI:1934811]

ENSMUSG00000043613	2.61734	0.000283	0.022292	Mmp3	matrix metallopeptidase 3 [Source:MGI Symbol;Acc:MGI:97010]
ENSMUSG00000033066	1.813053	0.000297	0.022917	Gas7	growth arrest specific 7 [Source:MGI Symbol;Acc:MGI:1202388]
ENSMUSG00000023191	1.335201	0.000296	0.022917	P3h3	prolyl 3-hydroxylase 3 [Source:MGI Symbol;Acc:MGI:1315208]
ENSMUSG00000074873	-1.97643	0.000295	0.022917	AI606181	expressed sequence AI606181 [Source:MGI Symbol;Acc:MGI:2147586]
ENSMUSG00000047414	2.203809	0.000302	0.023059	Flrt2	fibronectin leucine rich transmembrane protein 2 [Source:MGI Symbol;Acc:MGI:3603594]
ENSMUSG00000027188	1.741792	0.000303	0.023059	Pamr1	peptidase domain containing associated with muscle regeneration 1 [Source:MGI Symbol;Acc:MGI:2445082]
ENSMUSG00000026109	2.194588	0.000308	0.023202	Tmeff2	transmembrane protein with EGF-like and two follistatin-like domains 2 [Source:MGI Symbol;Acc:MGI:1861735]
ENSMUSG00000040552	2.169676	0.000307	0.023202	C3ar1	complement component 3a receptor 1 [Source:MGI Symbol;Acc:MGI:1097680]
ENSMUSG00000071984	2.989204	0.000316	0.023618	Fndc1	fibronectin type III domain containing 1 [Source:MGI Symbol;Acc:MGI:1915905]
ENSMUSG00000057123	1.349867	0.000325	0.024165	Gja5	gap junction protein, alpha 5 [Source:MGI Symbol;Acc:MGI:95716]
ENSMUSG00000039304	-0.69077	0.000334	0.024692	Tnfsf10	tumor necrosis factor (ligand) superfamily, member 10 [Source:MGI Symbol;Acc:MGI:107414]
ENSMUSG00000019278	1.732795	0.000379	0.02783	Dpep1	dipeptidase 1 (renal) [Source:MGI Symbol;Acc:MGI:94917]
ENSMUSG00000047497	2.128152	0.00039	0.028502	Adamts12	a disintegrin-like and metallopeptidase (reprolysin type) with thrombospondin type 1 motif, 12 [Source:MGI Symbol;Acc:MGI:2146046]
ENSMUSG00000026819	1.879951	0.000425	0.030646	Slc25a25	solute carrier family 25 (mitochondrial carrier, phosphate carrier), member 25 [Source:MGI Symbol;Acc:MGI:1915913]
ENSMUSG00000033207	1.62894	0.000423	0.030646	Mamdc2	MAM domain containing 2 [Source:MGI Symbol;Acc:MGI:1918988]
ENSMUSG00000028763	1.321204	0.000431	0.030939	Hspg2	perlecan (heparan sulfate proteoglycan 2) [Source:MGI Symbol;Acc:MGI:96257]
ENSMUSG00000031922	-1.04211	0.000464	0.033096	Cep57	centrosomal protein 57 [Source:MGI Symbol;Acc:MGI:1915551]
ENSMUSG00000090381	-1.07958	0.000476	0.03376	Gm6158	predicted gene 6158 [Source:MGI Symbol;Acc:MGI:3779562]
ENSMUSG00000032006	1.232254	0.000481	0.033924	Pdgfd	platelet-derived growth factor, D polypeptide [Source:MGI Symbol;Acc:MGI:1919035]
ENSMUSG00000087382	0.956693	0.000493	0.034561	Ctcflos	CCCTC-binding factor (zinc finger protein)-like, opposite strand [Source:MGI Symbol;Acc:MGI:1921411]
ENSMUSG00000038456	1.221939	0.000498	0.034694	Dennd2a	DENN/MADD domain containing 2A [Source:MGI Symbol;Acc:MGI:2444961]
ENSMUSG00000022098	1.593013	0.000502	0.03478	Bmp1	bone morphogenetic protein 1 [Source:MGI Symbol;Acc:MGI:88176]
ENSMUSG00000051323	1.507856	0.00052	0.035829	Pcdh19	protocadherin 19 [Source:MGI Symbol;Acc:MGI:2685563]
ENSMUSG00000038894	1.163727	0.000526	0.035989	Irs2	insulin receptor substrate 2 [Source:MGI Symbol;Acc:MGI:109334]
ENSMUSG00000024317	-0.90775	0.000528	0.035989	Rnf138	ring finger protein 138 [Source:MGI Symbol;Acc:MGI:1929211]
ENSMUSG00000031538	2.083476	0.00054	0.036603	Plat	plasminogen activator, tissue [Source:MGI Symbol;Acc:MGI:97610]

ENSMUSG00000022893	1.328471	0.000545	0.036758	Adamts1	a disintegrin-like and metallopeptidase (reprolysin type) with thrombospondin type 1 motif, 1 [Source:MGI Symbol;Acc:MGI:109249]
ENSMUSG00000028641	0.899637	0.000548	0.036773	P3h1	prolyl 3-hydroxylase 1 [Source:MGI Symbol;Acc:MGI:1888921]
ENSMUSG00000001930	1.928711	0.000565	0.037288	Vwf	Von Willebrand factor homolog [Source:MGI Symbol;Acc:MGI:98941]
ENSMUSG00000028664	1.392228	0.000564	0.037288	Ephb2	Eph receptor B2 [Source:MGI Symbol;Acc:MGI:99611]
ENSMUSG00000004642	-1.12055	0.000565	0.037288	Slbp	stem-loop binding protein [Source:MGI Symbol;Acc:MGI:108402]
ENSMUSG00000090084	2.045239	0.000573	0.037599	Srpx	sushi-repeat-containing protein [Source:MGI Symbol;Acc:MGI:1858306]
ENSMUSG00000032181	2.577384	0.000583	0.037839	Scg3	secretogranin III [Source:MGI Symbol;Acc:MGI:103032]
ENSMUSG00000017765	0.725287	0.00058	0.037839	Slc12a4	solute carrier family 12, member 4 [Source:MGI Symbol;Acc:MGI:1309465]
ENSMUSG00000031523	0.798592	0.000591	0.038156	Dlc1	deleted in liver cancer 1 [Source:MGI Symbol;Acc:MGI:1354949]
ENSMUSG00000023224	1.396351	0.000594	0.038157	Serping1	serine (or cysteine) peptidase inhibitor, clade G, member 1 [Source:MGI Symbol;Acc:MGI:894696]
ENSMUSG00000040711	1.822415	0.000608	0.038783	Sh3pxd2b	SH3 and PX domains 2B [Source:MGI Symbol;Acc:MGI:2442062]
ENSMUSG00000052727	1.693269	0.00061	0.038783	Map1b	microtubule-associated protein 1B [Source:MGI Symbol;Acc:MGI:1306778]
ENSMUSG00000026443	1.945743	0.000626	0.039604	Lrrn2	leucine rich repeat protein 2, neuronal [Source:MGI Symbol;Acc:MGI:106037]
ENSMUSG00000021268	2.145635	0.000639	0.039699	Meg3	maternally expressed 3 [Source:MGI Symbol;Acc:MGI:1202886]
ENSMUSG00000027386	1.990433	0.000637	0.039699	Fbln7	fibulin 7 [Source:MGI Symbol;Acc:MGI:1917620]
ENSMUSG00000028583	1.447742	0.000643	0.039699	Pdpn	podoplanin [Source:MGI Symbol;Acc:MGI:103098]
ENSMUSG00000045094	1.402908	0.000635	0.039699	Arhgef37	Rho guanine nucleotide exchange factor (GEF) 37 [Source:MGI Symbol;Acc:MGI:3045339]
ENSMUSG00000050002	-0.80725	0.000644	0.039699	Idnk	idnK gluconokinase homolog (E. coli) [Source:MGI Symbol;Acc:MGI:1922981]
ENSMUSG00000026580	1.647426	0.000666	0.04087	Selp	selectin, platelet [Source:MGI Symbol;Acc:MGI:98280]
ENSMUSG00000031486	1.23638	0.000697	0.042583	Adgra2	adhesion G protein-coupled receptor A2 [Source:MGI Symbol;Acc:MGI:1925810]
ENSMUSG00000014329	1.287122	0.000712	0.04326	Bicc1	bicaudal C homolog 1 (Drosophila) [Source:MGI Symbol;Acc:MGI:1933388]
ENSMUSG00000036446	2.09554	0.000739	0.044462	Lum	lumican [Source:MGI Symbol;Acc:MGI:109347]
ENSMUSG00000022096	1.309536	0.000736	0.044462	Hr	hairless [Source:MGI Symbol;Acc:MGI:96223]
ENSMUSG00000030022	1.001226	0.000752	0.045024	Adamts9	a disintegrin-like and metallopeptidase (with thrombospondin type 1 motif, 9 [Source:MGI Symbol;Acc:MGI:1916320]
ENSMUSG00000067071	-0.95864	0.000763	0.045468	Hes6	hairy and enhancer of split 6 [Source:MGI Symbol;Acc:MGI:1859852]
ENSMUSG00000001473	1.241074	0.00078	0.04623	Tubb6	tubulin, beta 6 class V [Source:MGI Symbol;Acc:MGI:1915201]
ENSMUSG00000006369	2.476519	0.000791	0.046506	Fbln1	fibulin 1 [Source:MGI Symbol;Acc:MGI:95487]

ENSMUSG00000030110	1.294324	0.000792	0.046506	Ret	ret proto-oncogene [Source:MGI Symbol;Acc:MGI:97902]
ENSMUSG00000028541	1.14673	0.000809	0.04727	B4galt2	UDP-Gal:betaGlcNAc beta 1,4- galactosyltransferase, polypeptide 2 [Source:MGI Symbol;Acc:MGI:1858493]
ENSMUSG00000031375	1.075354	0.000815	0.04727	Bgn	biglycan [Source:MGI Symbol;Acc:MGI:88158]
ENSMUSG00000031824	0.904635	0.000817	0.04727	6430548M08Rik	RIKEN cDNA 6430548M08 gene [Source:MGI Symbol;Acc:MGI:2443793]
ENSMUSG00000034675	1.288639	0.000853	0.049151	Dbn1	drebrin 1 [Source:MGI Symbol;Acc:MGI:1931838]
ENSMUSG00000043629	1.666067	0.000867	0.04946	1700019D03Rik	RIKEN cDNA 1700019D03 gene [Source:MGI Symbol;Acc:MGI:1914330]
ENSMUSG00000025722	-0.76345	0.000865	0.04946	Wdr73	WD repeat domain 73 [Source:MGI Symbol;Acc:MGI:1919218]
ENSMUSG00000024190	1.05063	0.000871	0.04951	Dusp1	dual specificity phosphatase 1 [Source:MGI Symbol;Acc:MGI:105120]
ENSMUSG00000027175	0.960828	0.000884	0.049817	Tcp11l1	t-complex 11 like 1 [Source:MGI Symbol;Acc:MGI:2444263]
ENSMUSG00000070738	-0.76921	0.000885	0.049817	Dgkd	diacylglycerol kinase, delta [Source:MGI Symbol;Acc:MGI:2138334]
ENSMUSG00000033705	1.062688	0.000899	0.04987	Stard9	START domain containing 9 [Source:MGI Symbol;Acc:MGI:3045258]
ENSMUSG00000026640	0.725771	0.000902	0.04987	Plxna2	plexin A2 [Source:MGI Symbol;Acc:MGI:107684]
ENSMUSG00000026238	-1.05222	0.000897	0.04987	Ptma	prothymosin alpha [Source:MGI Symbol;Acc:MGI:97803]
ENSMUSG00000027203	-1.12815	0.000899	0.04987	Dut	deoxyuridine triphosphatase [Source:MGI Symbol;Acc:MGI:1346051]

FC, fold change; FDR, false discovery rate

Supplementary data

Supplementary Table 5: Gene Ontology. Top 5 gene sets significantly changed in response to HFA for molecular processes, molecular function and cellular compartment ontology groups.

No	GO-ID	Term	Sig	Expected	FDR
		**Biological Process**			
1	GO:0040011	locomotion	145	44	3.50E-13
2	GO:0016477	cell migration	121	39	1.30E-12
3	GO:0048870	cell motility	129	40	2.70E-12
4	GO:0051674	localisation of cell	129	40	2.70E-12
5	GO:0006928	movement of cell	159	45	300E-12
		**Molecular Function**			
1	GO:0005518	collagen binding	17	10	7.80E-07
2	GO:0008237	metallopeptidase activity	18	10	1.60E-06
3	GO:0005539	glycosaminoglycan binding	27	12	3.00E-06
4	GO:0008201	heparin binding	23	10	2.70E-05
5	GO:0004222	metaloendopeptidase activity	12	7	4.50E-05
		**Cellular Compartment**			
1	GO:0005578	proteinaceous extracellular matrix	59	31	6.00E-17
2	GO:0031012	extracellular matrix	68	32	1.20E-15
3	GO:0005615	extracellular space	106	38	9.40E-14
4	GO:0005576	extracellular region	366	72	1.99E-10
5	GO:0044421	extracellular region part	344	66	4.00E-09

Supplementary data

Supplementary Table 6: GSEA (Hallmark) modulated by HFA diet. Top 10 enriched upregulated (+ NES) and down regulated gene sets (-NES) FDR 0 = < 0.001.

Hallmark Gene Set	Genes	En Score	NES	FDR
**Upregulated**				
Epithelial mesenchymal transition	164	7.848795	7.848795	0
Myogenesis	174	6.926522	6.926522	0
Apical junction	163	3.78442	3.78442	0
Estrogen response early	166	3.671612	3.671612	0
Kras signaling dn	83	3.348661	3.348661	0
Hypoxia	169	3.317838	3.317838	0
Coagulation	86	3.266531	3.266531	0
Uv response dn	132	3.114483	0.232409	0
Angiogenesis	27	3.047816	0.490317	0
Estrogen response late	153	2.973775	0.206369	0
**Downregulated**				
E2f targets	152	-0.46922	-6.67624	0
Myc targets v1	186	-0.3011	-4.81767	0
G2m checkpoint	153	-0.29988	-4.3706	0
Oxidative phosphorylation	190	-0.25466	-4.10262	0
Allograft rejection	115	-0.28414	-3.6242	0
Interferon gamma response	163	-0.24083	-3.6053	0
Dna repair	133	-0.2506	-3.32683	0
Myc targets v2	53	-0.24512	-2.12755	0.006921
Tnfa signalling via nfkb	151	-0.12676	-1.84845	0.027667
Interferon alpha response	84	-0.15811	-1.71064	0.049992

Supplementary data

Supplementary Table 7: GSEA (Hallmark EMT) modulated by HFA diet. Top 20 upregulated enriched gene sets.

PROBE	RANK IN GENE LIST	RANK METRIC SCORE	ES	CORE ENRICHMENT
Col6a2	5	7.99568	0.005684	Yes
Col6a3	8	7.37469	0.011616	Yes
Itga5	21	6.24109	0.016721	Yes
Ecm1	24	5.82102	0.022653	Yes
Pcolce	36	5.2644	0.02784	Yes
Col1a1	38	5.16941	0.033855	Yes
Fn1	40	5.16368	0.03987	Yes
Lama2	41	5.13549	0.045968	Yes
Col5a1	47	4.91364	0.051651	Yes
Loxl1	57	4.54821	0.057004	Yes
Loxl2	62	4.51856	0.062771	Yes
Col1a2	76	4.27901	0.067793	Yes
Thbs2	86	4.15864	0.073146	Yes
Timp3	91	4.11577	0.078913	Yes
Fbln5	98	4.03012	0.084514	Yes
Dcn	99	4.02872	0.090611	Yes
Col5a3	105	3.97455	0.096295	Yes
Fbn1	111	3.83255	0.101979	Yes
Col3a1	120	3.71768	0.107415	Yes

ES = Enrichment Score

Supplementary data

Supplementary Table 8: GSEA (Hallmark E2F Targets) modulated by HFA diet. Top 20 down regulated gene sets.

PROBE	RANK IN GENE LIST	RANK METRIC SCORE	ES	CORE ENRICHMENT
Stmn1	8358	-0.49184	-0.43453	Yes
Snrpb	8361	-0.4927	-0.4282	Yes
Smc1a	8509	-0.53234	-0.43386	Yes
Zw10	8526	-0.53471	-0.42869	Yes
Nop56	8594	-0.54969	-0.42773	Yes
Plk4	8595	-0.55044	-0.42124	Yes
Ran	8609	-0.55216	-0.41582	Yes
Rfc2	8694	-0.57462	-0.41627	Yes
Rnaseh2a	8727	-0.58748	-0.41242	Yes
Ncapd2	8734	-0.58904	-0.40642	Yes
Hn1	8848	-0.61681	-0.40927	Yes
H2afx	8888	-0.62744	-0.406	Yes
Pa2g4	8893	-0.62965	-0.39984	Yes
Pds5b	8934	-0.63875	-0.39665	Yes
Brca2	8947	-0.64236	-0.39115	Yes
Chek2	9046	-0.66685	-0.39276	Yes
Pnn	9088	-0.67969	-0.38965	Yes
Tfrc	9089	-0.67976	-0.38316	Yes
Dnmt1	9115	-0.68713	-0.37873	Yes
Dclre1b	9296	-0.73383	-0.38712	Yes

ES = Enrichment Score

Supplementary data

Supplementary Table 9: GSEA (C2) modulated by HFA diet**.** Top 10 enriched upregulated (+ NES) and down regulated gene sets (-NES) FDR 0 = < 0.001.

C2 Gene Set	Genes	En Score	NES	FDR
**Upregulated**				
Naba matrisome	429	0.490619	11.47848	0
Lim mammary stem cell up	367	0.479857	10.64709	0
Naba core matrisome	155	0.644179	9.129037	0
Liu prostate cancer dn	357	0.395048	8.824186	0
Boquest stem cell up	206	0.511573	8.38872	0
Naba ecm glycoproteins	113	0.619614	7.761062	0
Naba matrisome associated	274	0.402452	7.745561	0
Schuetz breast cancer ductal invasive up	279	0.398097	7.552965	0
Onder cdh1 targets 2 up	205	0.39839	6.732481	0
Cancer mesenchymal transition signature	52	0.761852	6.575606	0
**Downregulated**				
Pujana brca2 pcc network	322	-0.34389	-7.10385	0
Marson bound by e2f4 unstimulated	477	-0.25621	-6.24546	0
Reactome processing of capped intron containing pre mrna	126	-0.45432	-5.94819	0
Reactome metabolism of rna	212	-0.35828	-5.89353	0
Zheng bound by foxp3	368	-0.26897	-5.88158	0
Kegg spliceosome	112	-0.47993	-5.84562	0
Pujana xprss int network	138	-0.42321	-5.82428	0
Reactome mrna processing	143	-0.41927	-5.76118	0
Lee differentiating t lymphocyte	141	-0.4093	-5.58447	0

Supplementary data

Supplementary Table 10: Top Disease Pathways.

Disease and Disorders	P-Value Range	Number of Genes
Cancer	3.20E-04 – 6.30E-15	187
Organismal Injury and Abnormalities	2.99E-04 – 6.28E-12	219
Connective Tissue Disorders	3.18E-04 – 2.42E-11	139
Inflammatory Disease	5.12E-04 – 2.42E-11	205
Inflammatory Response	5.12E-04 – 6.85E-10	133

Supplementary Table 11: Top 5 Networks Identified from Ingenuity Pathway Analysis

Network Pathways	Score	Focus Molecules
Cell Morphology, Cellular Assembly and Organisation, Cellular Function and Maintenance	42	27
Cell Death and Survival, Cellular Movement, Hematological System, Development and Function	37	25
Cardiovascular Disease, Cardiovascular System Development and Function, Organ Morphology	21	17
Nervous System Development and Function, Organ Morphology, Tissue Morphology	19	16
Cancer, Organismal injury and abnormalities, tumour morphology	18	15

Supplementary data

Supplementary Table 12 Top Upstream Regulators.

Upstream Regulator	Molecule Type	Predicted Activation State	Activation Z Score	P-Value of Overlap
Alpha Catenin	Transcription Regulator	Inhibited	-4.502	1.39E-20
IL10RA	Transmembrane receptor	Activated	3.838	1.12E-12
IGF2BP1	Translation regulator	Activated	2.646	4.87E-11
COL4A3	Other			1.04E-09
FAS	Transmembrane receptor	Inhibited	-3.157	1.11E-09
IFNG	Cytokine		0.011	2.43E-08
TP53	Transcription Regulator		1.012	3.02E-08
RETNLB	Other	Activated	2.985	9.84E-08
NPR1	Enzyme	Inhibited	-2.415	2.16E-07
IL6R	Transmembrane receptor	Activated	2.219	3.78E-07

Supplementary data

Supplementary Table 13: Validation of differentially expressed Genes within RNA-seq data correlated to qRT-PCR data using Spearman's Correlation.

**Correlations**		***Mmp3* RNA-seq**	***Mmp3* qRT-PCR**
***Mmp3* RNA-seq**	Correlation Coefficient	1.000	0.879**
	Sig. (2-tailed)		0.001**
	N	10	10
***Mmp3* qRT-PCR**	Correlation Coefficient	0.879**	1.000
	Sig. (2-tailed)	0.001**	
	N	10	10

*. Correlation is significant at the 0.01 level (2-tailed).

**Correlations**		***Mmp2* RNA-seq**	***Mmp2* qRT-PCR**
***Mmp2* RNA-seq**	Correlation Coefficient	1.000	0.648*
	Sig. (2-tailed)		0.043*
	N	10	10
***Mmp2* qRT-PCR**	Correlation Coefficient	0.648*	1.000
	Sig. (2-tailed)	0.043*	
	N	10	10

*. Correlation is significant at the 0.05 level (2-tailed).

Supplementary data

Supplementary Table 14: Chip-seq enrichment analysis showing differentially expressed Genes within RNA-seq data that demonstrate an EZH2 binding site within the gene body or 500bp upstream/1000 bp downstream of TSS. *Hypergeometric Test; Benjamini-Hochberg.

Genes with EZH2 Binding Site

**500bp Upstream / 1000bp downstream TSS**	**Gene Body**
Cadm3	*Cdm3*
Col15a1	*Cd34*
Col6a2	*Col6a2*
Efhd1	*Fam124a*
Emilin2	*Fndc1*
Ephb2	*Gdf10*
Fam124a	*Hecw2*
Fndc1	*Itga11*
Gda	*Lama2*
Lama2	*Lg12*
Map1b	*Mapb1*
Mmp9	*Medag*
Npr1	*Npr1*
Sdk1	*Ptgis*
Tmeff2	*Sdk1*
Tspan11	*Tmeff2*
Tubb4a	*Tspan11*
Wnt2	*Tubb4a*
	Wnt2
**p = 7.30E^{-}05**	**p = 1.42E^{-}04**
